# Investigating negotiated treatment goals as a tool to facilitate collaboration between conventional and complementary practitioners in the treatment of People with Multiple Sclerosis

**Published:** 2011-09-27

**Authors:** Lasse Skovgaard

**Affiliations:** The Danish Multiple Sclerosis Society, Mosedalvej 15, 2500 Valby, Denmark; Department of Public Health, University of Copenhagen, Øster Farimagsgade 5, 1014 Copenhagen K, Denmark; National Research Center in Complementary and Alternative Medicine, NAFKAM, Faculty of Health Science, University of Tromsø, N – 9037 Tromsø, Norway

**Keywords:** multiple sclerosis, integrative treatment, integrative care, teambased treatment, complementary medicine, CAM, patient-centered health care, negotiated treatment goals

## Abstract

**Introduction:**

The Danish Multiple Sclerosis Society has conducted a research project where five conventional and five complementary practitioners as a team have treated people with Multiple Sclerosis (MS). As part of the treatment project's interdisciplinary framework, the team has worked with negotiated treatment goals. The overall purpose of this paper is to explore how negotiated treatment goals can contribute to an interdisciplinary treatment effort for people with MS.

**Theory:**

The study explores 1) How a flexible framework for involving negotiated treatment goals has been handled by the practitioners, 2) What potentials and challenges have been involved in the practitioners' work with negotiated treatment goals. The analyses are based on theories of epistemic cultures and learning theories, focusing on interdisciplinary development.

**Methods:**

The study was conducted as an exploratory case study. The data material consists of patient-owned records, referral schemes, questionnaires and completion reports.

**Results and conclusions:**

The flexible frame for the involvement of negotiated treatment goals has been handled in an incohesive way. Lack of time for coordination and trends for mono-professionalism as well as parallel treatment have posed challenges in the process, both for practitioners and patients. Despite these challenges, the team managed to implement successful treatment courses, not least due to a thorough pre-phase of collaboration.

**Discussions:**

The research results indicate that there is therapeutic potential in the use of negotiated treatment goals, but that a number of conditions must exist to obtain benefits from such negotiation.

11^th^ Annual Integrated Care Conference, Odense, Denmark, March 30–April 1 2011

